# Governance Options to Enhance Ecosystem Services in Cocoa, Soy, Tropical Timber and Palm Oil Value Chains

**DOI:** 10.1007/s00267-018-0996-7

**Published:** 2018-02-06

**Authors:** Verina Ingram, Jolanda van den Berg, Mark van Oorschot, Eric Arets, Lucas Judge

**Affiliations:** 1 Wageningen Economic Research, Wageningen UR, Wageningen, The Hague, The Netherlands; 2 Forest and Nature Conservation Group, Wageningen UR, Wageningen, The Hague, The Netherlands; 3 Dutch Environmental Assessment Agency (PBL), Den Haag, The Netherlands; 4 Wageningen UR, Wageningen, Alterra, The Netherlands

**Keywords:** Value chain governance, Tropical agricultural commodities, Ecosystem services, Integrated landscape approach

## Abstract

Dutch policies have advocated sustainable commodity value chains, which have implications for the landscapes from which these commodities originate. This study examines governance and policy options for sustainability in terms of how ecosystem services are addressed in cocoa, soy, tropical timber and palm oil value chains with Dutch links. A range of policies addressing ecosystem services were identified, from market governance (certification, payments for ecosystem services) to multi-actor platforms (roundtables) and public governance (policies and regulations). An analysis of policy narratives and interviews identified if and how ecosystem services are addressed within value chains and policies; how the concept has been incorporated into value chain governance; and which governance options are available. The Dutch government was found to take a steering but indirect role in all the cases, primarily through supporting, financing, facilitating and partnering policies. Interventions mainly from end-of-chain stakeholders located in processing and consumption countries resulted in new market governance, notably voluntary sustainability standards. These have been successful in creating awareness of some ecosystem services and bringing stakeholders together. However, they have not fully addressed all ecosystem services or stakeholders, thus failing to increase the sustainability of value chains or of the landscapes of origin. We argue that chains sourced in tropical landscapes may be governed more effectively for sustainability if voluntary, market policy tools and governance arrangements have more integrated goals that take account of sourcing landscapes and impacts along the entire value chain. Given the international nature of these commodities. These findings have significance for debates on public-private approaches to value chain and landscape governance.

## Introduction

Green economic growth requires embracing (and reconciling) national and cross-boundary policy issues such as climate change, poverty and equity, trade and value chains. As part of that agenda, the Dutch government has promoted considering ecosystems and ecosystem services generally in value chains for “enhanced biodiversity, water and food security, poverty alleviation and human well-being” (Netherlands Government 2013). This was in response to the European Union’s call as part of the Biodiversity Strategy to 2020 to its member states to map and assess the state of ecosystems and their services in their national territory (European Union 2013). Ecosystem services refer to the material and immaterial benefits humans derive from natural assets (Millennium Ecosystem Assessment 2005). These include provisioning services (also known as goods or products, such has food, fuel and water), and regulating, cultural and supporting services (CBD 2008). The provision of ecosystem services is culturally determined, conceptualized as the “useful things” ecosystems “do” for people, directly and indirectly, and so is dynamic, changing over time even if the ecological system itself remains in a relatively constant state (TEEB 2010).

The Netherlands has a history of promoting sustainable trade and safeguarding healthy social and environmental conditions in the sourcing regions of imported goods. It has sought to increase the sustainability of international trade using a value chain approach, as part of a long-term transition of Dutch trade, development and environmental policy toward a green economic growth paradigm (Keijzers 2000; Elzen et al. 2004). The Dutch government has used four main governance approaches to implement this policy: mandating, partnering, facilitating, and endorsing. The corresponding governance arrangements can be found in Table 1 and include government regulation, closed co-governance, open co-governance facilitating self-regulation, and market governance respectively (van Tulder 2008; Vermeulen and Kok 2012) (see next section for further details).

**Table 1:** Governance arrangements and policy instruments in value chains

Governance arrangement				
Role of government:	Mandating:	Partnering:	Facilitating:	Endorsing:
	Government regulation	Closed co-governance	Open co-governance	Market governance
Policy instruments and interventions:	Coercion, ‘command and control’ legislation, regulators and inspectors, legal and fiscal penalties, payments e.g., transfer payments and grants, tax regimes, public labels & standards, anti-trust rules, policies, direct action	Combining resources, actors engagement, dialog, public private partnerships, covenants/agreements	'Enabling legislation’, actor dialog, awareness raising, incentives, subsidies, tax rebates, procurement policies, capacity building, supporting spread of labels, self-governing agencies	Product labeling, support for/by civil society initiatives, Industry ‘Best practices’, voluntary labeling and certification standards
Corporate governance Codes:	Stock exchange regulations and codes, company law, mandatory reporting, disclosure rules	Multi-actor code development, shared monitoring of government, market or civil society initiated or shared incentives	Implementing international principles, reporting stimuli/guidelines, internalization, incentives	Own responsibility: civil society and market initiated, voluntary codes and reporting; peer reviews/pressure

Inspired by van Tulder (2008) and Vermeulen and Kok (2012)

With a view to reducing the impact on the sourcing areas from where the value chains originate and securing resilient ecosystems, the Dutch government (2013) has integrated the concept of environmental services in its value chain approach. The ecosystem services concept is seen as capable of bridging natural and social sciences and economics, conservation and development, and public and private policy (Braat and de Groot 2012). Making ecosystem services more visible by assigning economic value to them can inform decision-makers about the importance of ecosystem services and change perceptions on economic development and the future of the globe (Costanza et al. 1997 and 2017; TEEB 2009). The ecosystem services concept helps understand how businesses affect natural capital (TEEB 2009) and thus can be used to examine the sustainability of value chains. The increasing use of this concept reflects a paradigm shift both in the Netherlands and internationally (van Wensem 2013; Wittmer et al. 2013), indicated by phrases such as “making natures value’s visible”, “mainstreaming nature” and “valuing natural capital” (TEEB 2009; Melman et al. 2011). Sustainable ecosystem services imply that despite the services or products of an ecosystem being used, the integrity and proper functioning of its natural processes and components is not irreversibility impaired (De Groot et al. 2002). This is a critical point as losses of global land-based ecosystem services have been valued at around € 50 billion annually (TEEB 2009). Ecosystem losses have important implications for the long-term viability of businesses and value chains dependent upon the supply of ecosystem services and/or products originating from these ecosystems (TEEB 2009). Integrating ecosystem service management in value chain governance assumedly therefore results in different benefits for different stakeholders. These include, first, private benefits for resource producers, by making businesses more viable—for instance through the commodification of ecosystem services. Second, there are public benefits for people living in and nearby sourcing areas who are dependent upon an ecosystem’s products and services, by increasing the sustainability of their livelihoods. Third, there are global societal benefits such as climate regulation and biodiversity conservation.

Some of the impacts of sustainable value chains are only visible when a landscape perspective is taken. By reducing environmental externalities, sustainable value chain management creates both on-farm and in-forest benefits and reduced costs for the farmers, harvesters and broader landscape stakeholders. To capture such wider impacts, governance arrangements need to go “beyond the chain” (Ros-Tonen et al. 2015) and to tackle the sustainability of ecosystem services at the landscape scale (Muller et al. 2010). Often collaboration between value chain and non-chain actors is needed, as such ‘advanced value chain collaboration’ can bring a greater positive impact on farmers’ social, human and natural capital than conventional value chain collaboration (Deans et al. 2017). The landscapes where soy, palm oil, timber and cocoa are produced for the Netherlands are largely tropical low- and middle-income countries. The exception is soy, which is sourced mainly from Brazil, the USA, Argentina, China, India and Paraguay (WWF 2014). Source impacts from commodity production often include deforestation, degradation and associated biodiversity loss; climate and environmental impacts such as over-use and pollution of water; and socio-economic issues such as low wages and forced labor. These impacts are further exacerbated by political instability and weak state governments (c.f. van den Berg et al. 2013; van den Berg et al. 2014).

There are several challenges to maintaining and enhancing ecosystem services impacted by commodity trade. First, the range of ecosystem services are often addressed by different decision-making processes and policies, such as forestry agencies, government bodies responsible for land-use planning, and environmental ministries (van Oorschot et al. 2016). Second, terminology is varied, with the terms “ecosystem services” and “landscape functions” often referring to the same underlying concepts, due to the diversity of disciplinary backgrounds behind these transdisciplinary concepts (Muller et al. 2010; Arts et al. 2017). Third, contrasting discourses and often sectorally defined policy frames view commodity value chains, their origin landscapes and associated ecosystems differently (van Oosten et al. 2017). Against this context, this paper takes a broad perspective on the relations between ecosystem services, the biophysical characteristics of landscapes and their products, and subsequent value chains. It thereby addresses the following questions:

1. How have ecosystem services been positioned in Dutch policies from 2007 to 2014 to increase the sustainability of international value chains with links to the Netherlands?
2. How are ecosystem services incorporated into the Dutch-linked value chains?
3. Which governance options are available to increase the sustainability of international value chains by addressing ecosystem services?

These questions are addressed in the results section, after outlining the conceptual framework that guides the analysis, and the methodology used. The discussion deliberates on the need to expand the value chain approach to a broader landscape perspective that goes “beyond the chain” (Ros-Tonen et al. 2015).

## Conceptual Framework: Value Chain Governance

To guide this study, a framework drawing on value chain governance concepts was constructed. Value chains concern the value-generating activities involved in bringing a product—farmed and natural—from its origins, through processing and production, to delivery to final consumers and ultimately disposal (Kaplinsky and Morris 2000). These activities may be implemented by various actors, including primary producers and harvesters, processors, traders, service providers, and upstream suppliers. Products embody and carry with them multiple relations of value – often explicitly economic, but also social, cultural and environmental (Ingram 2014). Value chains are dynamic and diverse and can operate from local to national and global level, with international chains connecting the origin landscapes where products are sourced to those in which processing and consumers are embedded, with positive and negative impacts possible at all stages of the chain. From this perspective, a landscape coincides with the sourcing area of a value chain (c.f. Ros-Tonen et al. 2015; Deans et al. 2017). Value chain analysis provides a framework for mapping and categorizing the interactions, relationships and power between chain actors and the economic, social and environmental processes in chains, to create a better understanding of how and where actors are positioned and benefit or lose out. Value chains encompass the organization, coordination and linkages, power dynamics, and governance between actors (Helmsing and Vellema 2011) and as such can be used to investigate governance and identify opportunities and possible leverage points for interventions and changes in chain arrangements (Humphrey and Schmitz 2001).

‘Governance’ is central in value chains, referring to the relationships and institutional mechanisms through which the coordination of activities in a chain take place (Humphrey and Schmitz 2001) and the relative powers between stakeholders in a chain (FAO 2007; Keane 2008). Institutions enable and shape individual, group and social expectations, interactions and behavior through the rules, norms and processes that define how people interrelate and act within and outside of organizations (UNDP 1997; Bavinck et al. 2005). Institutions can change over time or space, be formal or informal, and are interlinked with knowledge, power and control. Institutions may govern ecosystem services (van Oorschot et al. 2016), including provisioning ones that generate agricultural and natural commodities. Governance arrangements can be seen on continuum, shown in Table 1, which vary depending upon the goals and actors (van Tulder 2008; Vermeulen and Kok 2012). Government regulation focuses primarily on public goals, whereas in closed co-governance, a coalition of (usually) government and private sector adopts public goals. In open co-governance public goals are negotiated, with government facilitating self-governance. Self-governance concerns common goals scaled-up to become public goals or coupled to them (Fernandez-Stark et al. 2011; Arnouts et al. 2012). Market governance refers to public aims being coupled with business interests. These new and hybrid forms of governance can occur in alliances between public, private and civil society actors.

Coordination in value chains is achieved through the setting and enforcement of product and process conditions to be met by stakeholders in a chain, for example through networks, and platforms. In international value chains, buyers often play an important role in setting and enforcing such conditions because a (perceived) risk of producer failure (Humphrey and Schmitz 2001). Product and process conditions and standards may also be set by government agencies and international organizations, such as environmental standards which may address ecosystem services. Value chains are subject to increased complexity, proliferating jurisdictions, multiple centers of decision-making in government and non-state realms, and the increased rise and participation of non-chain actors in international value chains. This gives rise to notions such as multilevel and polycentric governance. These phenomena make decision-making a process of “complex overlapping networks” (i.e., governance institutions) at multiple scales, rather than “discrete territorial levels” (Bache and Flinders 2004). Changes in the role of the government and firms have challenged conventional ideas of democratic accountability and altered their roles in decision-making, corporate social responsibility and transparency in value chain management Fernandez-Stark et al. 2011; (Arnouts et al. 2012). This has led to four main types of policy instruments currently being used to address value chain activities (Table 1).

This paper focuses on the integration and maintenance of ecosystem services into value chain governance and policy against the broader framework conditions—the meso and macroeconomic context and landscapes in which value chains operate. These include the socio-economic, regulatory, institutional and political environment; market demand and consumer characteristics and trends; the direct business operating environment; the structure and composition of production systems; and the wider political system. These framework conditions provide “windows of opportunity” for interventions concerning ecosystem services in value chains.

## Methods

First, a review of policy documents, scientific literature and websites from 2007 to 2017 was used to identify public and private policies addressing ecosystem services in Dutch-based commodity value chains. The documents resulting from this review are shown in Table 2. Framing (Hanke et al. 2002) was used to identify how Dutch policy makes sense of stakeholders, their roles and relationships. A frame provides a link between the messages in the literature that may or may not be deliberately provided, to broader perceptions about the world around us (Gorp 2006). The main frames were constructed from the content and keywords (ecosystem services and the specific services named in the Millennium Ecosystem Assessment 2005, ecosystem(s) approach, valuing, natural capital) occurring in the literature reviewed.

**Table 2:** Dutch policy documents concerning ecosystem services and value chains

Responses to policy, evaluations and advice:
1. Letter from the Ministry of EA in response to the advice of the Taskforce Biodiversity and Natural Resources (Ministry of Economic Affairs, Agriculture and Innovation 2012)
2. Letter of appreciation of the Ministry of EA concerning the European Biodiversity Strategy (Ministry of Economic Affairs, Agriculture and Innovation 2011)
3. Assessing IDH’s contribution to public good impacts at scale (2016–2020). First assessment report on the existing evidence behind IDH’s impact stories. Wageningen, Wageningen University & Research and KPMG Advisory N.V.: 121. (Waarts. Y and K. Basso Gumbis de souza 2017)
Policy documents addressing value chains and/or ecosystem services:
4. Government Commodity Note (Dutch Cabinet 2011)
5. Government Sustainability Agenda. A green growth strategy for the Netherlands (Ministry of Infrastructure and Environment 2011)
6. Biodiversity Policy 2008–2011. Biodiversity works for nature for people forever (Ministry of Agriculture, Nature and Food Quality 2012)
7. Natural Capital Agenda: Conservation and sustainable use of biodiversity (*Uitvoeringsagenda Natuurlijk Kapitaal: behoud en duurzaam gebruik van biodiversiteit*) (Ministry of Economic Affairs 2013)
8. Policy Letter. Corporate social responsibility pays off (Ministry of Foreign Affairs 2013a)
9. Policy Note. What the world deserves: a new agenda for aid, trade and investment (Ministry of Foreign Affairs 2013b)
10. Report Dutch international support in the field of climate change (Ministry of Foreign Affairs 2010)
11. Sustainable Trade Action Plan 2011–2015. Public-private partnership for sustainable commodity chains (IDH 2012)
12. 2016–2020 Strategy. Innovating for impact @ scale. IDH next stage of sustainable supply chain interventions. IDH, The Sustainable Trade Initiative (IDH 2016)
Policy documents on governance and collaboration:
13. Background document for the budget of the Ministry of EA 2011 (Dutch House of Representatives 2011)
14.Government vision on governance and administrative structure (Ministry of the Interior and Kingdom Relations 2011).

Second, of the seven priority commodity value chains addressed in the Dutch Sustainable Trade Action Plan (IDH 2012), four (tropical timber, cocoa, soy and palm oil) were selected for study. The high levels of imports of these raw and processed products to the Netherlands, processing and (re)exports have created a strong Dutch economic and political interest in these chains. Thirdly, a review of scientific literature, websites, databases and media from 2007 to 2015 was used to identify cases of how ecosystem services were addressed in these value chains. Fourthly, additional information on the cases was gathered through interviews with 25 people involved in these value chains, working with the private sector, voluntary certification schemes, government, NGOs, research organizations and consultants. The interviews did not aim to provide a representative perspective from all parties in the chain. For this reason the interviews are anonymous. As actors can also be information gatekeepers, semi-structured questions were used to avoid bias and triangulate the data and focus on if and how ecosystem services were positioned in Dutch policies, how ecosystem services were incorporated into value chain governance (where, how, by whom, and the relationships between actors) and the governance options available to increase the sustainability of international value chains by addressing ecosystem services.

## Results

This section presents the results according to the three research questions

1. How have ecosystem services been positioned in Dutch policies from 2007 to 2014 to increase the sustainability of international value chains with links with to the Netherlands?
  Dutch policies referring to value chains and governance consistently lack a definition of ecosystem services and address ecosystem services mostly implicitly, as one element of a wider objective (sustainability), often defined selectively by different policy instruments. Of the policy documents, only the Natural Capital Agenda (Netherlands Government 2013) explicitly concerns ecosystems services and relates these to conservation and the sustainable use of biodiversity, with a link made between natural capital and value chain sustainability. By 2014, the focus shifted from making biodiversity and other ecosystem services more concrete (De Knegt 2014; Smits et al. 2013) to the concept of natural capital, which is arguably more accessible and applicable (Kok et al. 2014). This led to ecosystem services being positioned in Dutch policy as both a national, European and global issue.
  Ecosystem services are positioned in different governance arrangements. The main policy document providing the most extensive information on ecosystem services is the 2008–2011 Biodiversity policy—the oldest of the documents—where ecosystem services are introduced with reference to the Millennium Ecosystem Assessment. Ecosystem services were predominantly associated with markets and payment mechanisms, while biodiversity was associated with sustainable trade chains. The policies strongly emphasize markets and stimulate attempts to define the economic value of ecosystem services. The policy documents suggest that the main cause of ecosystem degradation is due to the costs of losing biodiversity and ecosystem services not having market prices. This externalization of ecosystem costs resulted in attempts to define the economic value of ecosystem services so that they can be internalized, at least partly, into chain activities. This led to a focus on economic policy measures such as the Sustainable Trade Initiative (*Initiatief Duurzaame Handel*, IDH), marketed as a public-private partnership, supported by government financing; a closed co-governance solution to conserve and maintain ecosystem services. Sustainability challenges for business were framed as an opportunity to strengthen the competitive position of the Netherlands, particularly in recent policy documents.
  The second dominant frame concerns the distribution of responsibilities and the need for cross-sector collaboration and partnerships between government, industry, research and civil society and the government taking a supporting and facilitating role. In line with this open co-governance response, the government has invested in the development of multi-actor platforms and collaborations and in developing a policy agenda which further integrates economy and ecology, and in which businesses take the main or leading role in developing sustainable value chains. Dutch development cooperation policy shifted “from aid to trade”, which coincided with the joint plea from civil society organizations, private business and trade unions for a concerted, long-term sustainability agenda for Dutch international trade. Demand was created and stimulated for (certified) standards which demonstrated the sustainability credentials of products. These developments implied a change in the framework conditions in which the value chains operated.
  The third frame relates to market governance, with certification as one of the main mechanisms used to promote more sustainable chains. It has been the main approach in which ecosystem services were either specifically named or implied. Among the voluntary sustainability standards promoted as tools to implement the Dutch policy, only the Forest Stewardship Council (FSC) and Forest Certification for Ecosystem Services (ForCES) certification explicitly mention ecosystem services. The certification standards used in the cocoa, soy and palm oil chains mention only specific services such as genetic resources, erosion regulation and water quality, but do not use the term ecosystem services. Experiments with the use of payments for ecosystems (PES) in the cocoa chain and reducing emissions from deforestation and forest degradation (REDD+) in the timber chain are the only other examples of market-led policy instruments which explicitly allude to ecosystem services.
  The 2010–2015 Sustainable Trade Action Plan (IDH 2012) discussed neither ecosystem services nor value chain governance, but 5 years later in the 2015–2020 strategy (IDH 2016), governance was seen as an important pathway to develop sustainable commodity chains. However, the ecosystem services related to these chains were not made explicit. In the impact evaluation of the Sustainable Trade Initiative (Waarts and Basso Gumbis de Souza 2017), both value chains and landscape governance were seen as key pathways contributing to impact and ecosystem impacts of commodity trade. Natural capital and ecosystem services were not specifically mentioned, but ecosystem health and impacts on ecosystems were. However, the plausibility and impacts of the IDH approach to improving sector and value chain governance has been noted as difficult to assess, given the limited amount of information available (Waarts and Basso Gumbis de Souza 2017).
  The Dutch policies to increase the sustainability of commodity value chains mirror global trends. Sustainability policies have been implemented by the UNDP Green Commodity Program since 2009 in eleven countries concerning the palm oil, cocoa, coffee, pineapple, fisheries, soy and beef chains (UNDP 2017) and in Germany (Eberhard Krain and Edmond Konan 2011; FAO 2007), Switzerland (Auroi 2003; Hamprecht et al. 2005; Schouten and Glasbergen 2011; Vermeulen and Kok 2012), the UK (Walker and Jones 2012) and Denmark (Giovannucci et al. 2014). This convergence of policies is reflected in increasing co-funding of the IDH: originally solely Dutch-funded it is now also financed by the Swiss, Danish and Norwegian governments.
2. How are ecosystem services incorporated into the Dutch-linked value chains?
  This section successively provides an overview of the eight cases that address ecosystem services in the four value chains, the actors driving the change, and where in the chain and how ecosystem services were addressed (Table 3); how the eight cases have integrated ecosystem services in the value chains (Table 4); and which actors are involved in the cases (Table 5). Notable are the similarities: a focus on process-orientated, multi-stakeholder platforms and partnerships, and on the producer stage at the beginning of the chain. The process-oriented pilots general entailed IDH match funding companies to help develop, implement and scale-up certification schemes. The cases show that interwoven technical, process- and learning-orientated organizational, economic and institutional measures were used in parallel to address sustainability issues in all four value chains. In five of the cases—UTZ, PES, Roundtable for Responsible Soy (RTRS), FSC and the Roundtable for Sustainable Palm Oil (RSPO)—specific rather than all ecosystem system services were addressed; in two cases (STAP and the Dutch Procurement Policy), ecosystem services are implicitly addressed through reference to certification standards; and in three cases (FSC, ForCES and REDD+) ecosystem services were specifically mentioned (Table 3).

**Table 3:** How ecosystem services were addressed in the tropical timber, cocoa, soy and palm oil value chain cases

Chain	Case	Driving stakeholder	Value chain stage where ES addressed	Ecosystem services (ES)	Characterization
Cocoa	Sustainable Trade Action Plan (STAP) 2010 & UTZ certification 2008	Private sector (international and Dutch)	Whole chain, particularly producers	ES not explicit in STAP but some ES addressed in UTZ standard	Process-orientated pilot, multi-actor platform
	Payments for ES (PES) 2010	Private sector (Dutch)	Whole chain, particularly producers	Payments for specific ES	Pilots Ghana & Côte d’Ivoire
Soy	Roundtable for Responsible Soy (RTRS) 2006	Private sector (international & Dutch), civil society, public sector	Whole chain, particularly producers	Attempts to include some ES in the RTRS standard	Process-orientated pilot, multi-actor platform
Palm oil	Roundtable for Sustainable Palm Oil (RSPO) 2003	Private sector (international & Dutch)	Whole chain, particularly producers	Some ES addressed in RSPO standard	Process-orientated pilot, multi-actor platform
Timber	Sustainable Trade Action Plan (STAP) 2011	Dutch government	Exporters, manufacturers, retailers	ES implicit via use of voluntary sustainability certification standards	Multi-actor partnerships and platform, finance
	Forest Stewardship Council (FSC) 1993 & Forest Certification for Ecosystem Services (ForCES) 2012	Private sector (international & Dutch)	Whole chain, particularly forest owners/concessionaires	ES addressed in the FSC standard; ForCES certifies ES	Process-orientated pilot, multi-actor platform
	Dutch Public Procurement Policy 2008	EU & Dutch government	Whole chain, particularly importers, end buyers and users	ES implicit by referring to FSC and PEFC certification standards	Product- and process- orientated policy; GFTN and TPAC as multi-actor platforms; regulations on sustainability standards in chains
	Reducing Emissions from Deforestation & Forest Degradation (REDD + ) Indonesia 2010	International and Dutch NGOs, United Nations, Dutch government	Particularly forest owners/concessionaires, private sector	REDD + specifically mentions ES	Multi-actor platform and partnership, policy practice & research, pilots, learning-orientated, resource-focused

**Table 4:** How the ecosystem services were addressed in the four value chain cases

Strategy/instrument	Cocoa		Soy	Palm oil	Timber			
	IDH & UTZ	PES	RTRS	RSPO	IDH	FSC & ForCES	Dutch Public Procurement Policy	REDD+
Introducing and upscaling voluntary certification standards	√		√	√	√	√	√	√
Partnering and partnerships, including platforms	√	√	√	√	√	√	√	√
Promoting an enabling environment for ecosystem services	√		√	√	√	√	√	√
Simplifying access to information	√		√	√		√	√	
Encouraging entrepreneurship	√		√	√	√		√	
Recognizing the role of intermediaries	√				√		√	
Enhancing and supporting collective action	√				√	√		
Commodity innovation		√				√		√
Creating and testing positive cases and situations and building on experiences								√
Regulation							√

**Table 5:** Overview of actors engaged in the value chain cases

Case	Dutch Government	Government production (countries)	NGOs/CSOs	Research	Private sector	Other
Cocoa certification	Ministries of Economic and Foreign Affairs	Indirectly through commodity programs & projects	Involved in IDH STAP as partners e.g., Solidaridad	Indirectly through monitoring and evaluation studies	Traders and processing companies, wholesalers and retailers	Certification and support organizations e.g., UTZ Certified
Cocoa PES	Ministry of Economic Affairs	Directly through projects in origin countries e.g., Ghana	AgroEco Louis Bolk	–	Consultants, traders and processing companies	–
Soy RTRS	Ministries of Economic and Foreign Affairs	Indirectly through commodity programs & projects	Involved directly through membership of RTRS, via IDH STAP, funding projects and evaluations	Indirectly through monitoring and evaluation studies	Traders and processing companies, wholesalers and retailers	RTRS Secretariat, certification, audit and support organizations
Palm oil RSPO	Ministries of Economic and Foreign Affairs	Indirectly through commodity programs & projects	Involved in IDH STAP, RSPO, directly initiators e.g., WWF, funding projects and evaluations	Indirectly through monitoring and evaluation studies	Traders and processing companies, wholesalers and retailers	RSPO Secretariat certification, audit and support organizations
Timber IDH-STAP	Ministry of Foreign Affairs	Indirectly through commodity programs	Involved in STAP as partners e.g., WWF	Indirectly through monitoring and evaluation studies	Dutch concession holders & processing companies, wholesalers and retailers	FSC Netherlands and support organizations
Timber FSC & ForCES	Indirectly (Ministry of Foreign Affairs and other bilateral aid agencies) potential buyers	Indirectly through FLEGT/VPAs	Very active (ForCES: WWF,SNV, RECOFTC/ FSC, WWF, Greenpeace, SMN, ICCO)	Directly via CIFOR	FSC: concession holders & timber companies.	UN, GEF, FSC national and international, ANSAB
					ForCES: forest managers, concession holders, private sector	
					ES buyers not identified	
Timber Dutch public procurement	Cabinet, Parliament, Ministry of Infrastructure & Environment	Indirectly through TPAC actor internet forum	Indirectly via TPAC internet forum & watchdogs (Friends of the Earth NL, Greenpeace, ICCO, WWF)	–	Building contractors, timber industry, timber importers, (VVNH)	Stichting Probos, Centrum Hout, Stichting Milieukeur, TPAC actor internet forum, AgentschapNL, FSC-NL, PEFC- NL, PIANOo, TPAC
Timber REDD +	Ministries of Economic and Foreign Affairs	National governments	Indirectly through advisers, consultants, conducting studies	Indirectly through advisers, conducting studies	–	UN, World Bank

Translations and abbreviations from Dutch *(*in *italics)*

*AgentschapNL* Dutch Agency, *Stichting Probos*, Probos Association, *Centrum Hout* Timber Centre, *Stichting Milieukeur* Environmental Certification System Association, *ANSAB* Asia Network for Sustainable Agriculture and Bioresources, *CIFOR* Centre for International Forestry Research, *FLEGT* Forest Law Enforcement and Governance and Trade, *FSC* Forest Stewardship Council, *ICCO* International Cocoa Organisation, *IDH* Sustainable Trade Initiative, *PEFC* Program for the Endorsement of Forest Certification, *PIANOo* Expertise centre for Contracting, Dutch ministry of Economic Affairs, *RECOFTC* Centre for People and Forests, *REDD* Reducing Emissions from Deforestation and Forest Degradation, *RSPO* Roundtable on Sustainable Palm Oil, *RTRS* Roundtable for Responsible Soy, *SMN* Stichting Natuur & Mileu Association for the Environment & Nature, *SNV* Netherlands Development Organization, *STAP* Sustainable Trade Action Plan, *TPAC* Timber Procurement Assessment Committee, *UN* United Nations, *VPA* Voluntary Partnership Agreement, *WWF* Worldwide Fund for Nature, *VVNH* Netherlands Timber Trade Association
  Table 4 provides an overview of how the value chain cases address sustainability and integrate ecosystem services in the value chains. The most popular strategies and instruments were certification, partnering, and promoting an enabling environment for ecosystem services by raising awareness of ecosystem services as a means to develop new business practices. Most strategies and instruments were framed as being along the entire value chain, but were in fact focused on the producer stage and on setting up platforms and networks of value chain actors. The public procurement policy in the timber chain was the only case based on statutory regulations.
  Multi-stakeholder involvement and partnerships between private sector, non-government and civil society organizations, and research institutes were the most common approach to trigger and implement changes in chain governance, with examples found in all the chains (Table 5). The majority of interviewees considered multi-stakeholder partnerships as critical to the success of setting up and implementing the new arrangements. The majority of the Dutch private sector chain participants were large multi-nationals, with few examples of small and medium enterprises. The Dutch government largely played an indirect facilitating and endorsing role. There was no or minimal liaison with governments in origin countries. In the cases involving cocoa certification, soy and palm oil, IDH and the main private sector actors implement Dutch policy, with the government funding, steering and evaluating this implementation. The participation of civil society was generally limited and consumers (private, corporate and public) were largely absent in the development and direct implementation of policies, except in the timber public procurement regulation. Consumers were however stimulated to change their purchasing behavior through the use of certified products.
3. Which governance options are available to increase the sustainability of international value chains by addressing ecosystem services?

The cases in the previous sections show that a range of governance arrangements and policy approaches have been used in tropical commodity value chains ending in the Netherlands. The main policy approaches used have been endorsing, partnering and facilitating. Both government and non-government actors were incorporated into new governance arrangements used to stimulate and support changes toward more sustainable chains, with ecosystem services mostly implicitly, except for four cases (UTZ cocoa certification, soy RTRS, palm RSPO, FSC timber certification) and explicitly addressed. Initially, pressure from civil society played a major role in developing more sustainable value chain practices. Companies and NGOs often worked together in establishing and defining production standards (van Tulder 2008; Vermeulen and Kok 2012; van den Berg et al. 2013, 2014). Market governance, notably certification, has been the dominant approach to increase the sustainability of the four commodity value chains. Considering the limited market share and long adoption timescales, interviewees questioned whether voluntary mechanisms are being implemented fast enough to meet all the challenges inherent in making value chains more sustainable. Although the success of FSC certification has been much lauded (Synnott 2005; van Kuijk et al. 2009; Oldenburger et al. 2010), with FSC-certified forests covering 12% of all tropical forests in 2011 (Forest Stewardship Council 2012), only around 0.4% of global tropical roundwood production is certified (UNECE/FAO 2011). FSC certification is much lower for tropical and subtropical biomes with 11.5% of the total forest area certified, compared to 52% of boreal and 37% of temperate biomes (Forest Stewardship Council 2012). The adoption of certified cocoa has been faster, with approximately 38% of global production certified since 2008 (Fountain and Hutz-Adams 2015), and 25% of cocoa sold on the Dutch market being certified (Logatcheva 2014). About 41% of palm oil sold in the Netherlands is certified, largely since 2010 (CBS 2013) and since 2010 approximately 8% of soy sold on the Dutch market is certified (CBS 2013). This progress has prompted interest in going “beyond certification” (Barry 2015; Poynton 2015), and “certification plus”: certification accompanied by other initiatives such as capacity building, training of producers and policies, which is becoming increasingly common, particularly for commodities with longer histories of certification such as cocoa (Ingram et al. 2017; Deans et al. 2017). The difficulties of measuring impacts of commodity chain interventions and governance (Waarts and Basso Gumbis de Souza 2017) have also highlighted the need for integrated, performance-based incentive systems operating across regions and scales, linked through a shared metric of jurisdiction-wide performance (Nepstad et al. 2013).

Some interviewees (notably CSOs and some private sector actors) raised questions about whether framework conditions were addressed considering the indirect role of the Dutch government and limited involvement of governments of countries of origin. It was noted that the reliance on market governance using certification standards, meant that impacts are most prevalent in origin landscapes, but this is where the Dutch government and its agencies have little authority.

A third of the interviewees noted that a stronger combination of both statutory “command and control” and voluntary instruments would allow higher standards to be developed through voluntary mechanisms. Regulations would enable minimum standards to be set to ensure complete chain and sector coverage, which is currently lacking (Waarts and Basso Gumbis de Souza 2017). An example is the development of the ISO Sustainable Cocoa Standard, with the Dutch government participating in the concurrent Dutch Sustainable Cocoa Norm. Mirroring these sentiments, Lambin and colleagues 2014 note that public regulations also play a role in providing enabling conditions for market and hybrid governance initiatives, pushing standards upward, and are critical in some framework contexts, such as controlling for weak governance. Market governance however has the potential to address regulatory gaps and improve land-use practices and contribute to broader changes in governance, under appropriate policy mixes.

The responses of actors and the evaluation of IDH (Waarts and Basso Gumbis de Souza 2017) indicate that a wider range of policies and arrangements in both origin and consumer countries is needed, and that both consumer and processing country governments need to be involved. Maintaining ecosystem services related to commodities and their value chains needs to go beyond the farm or forest from where these commodities are sourced, and cannot be isolated from the broader landscapes from which they originate. Complementary policies and governance approaches are needed to spur a transformation toward more sustainable trade and upscale the adoption and acceptance of sustainability initiatives in value chains, recognizing the limits of voluntary chain-based initiatives to involve or stimulate all market actors (Oorschot et al. 2013). The discussion below deliberates on whether landscape approaches could move value chain interventions beyond certification, with policies and instruments that address ecosystem services in the commodity origin landscape in a more integrated way.

## Discussion: Going beyond the Chain and Integrating Landscapes into Value Chain Governance Arrangements

Given the international nature of the four commodity value chains, lessons from Dutch policies have significance for debates on government-market/public-private approaches to value chain and landscape governance. Concerns to maintain ecosystem services in relation to commodities have occurred globally, as have the commodification of ecosystem services, with companies and civil society organizations promoting new business models and approaches such as trading individual, segregated ecosystem services in specialist or niche markets, such as carbon (Bishop et al. 2009), bundling ecosystems services (Renard et al. 2015), and payments for ecosystem services (PES) via certification (Wunder 2006; Felperlaan et al. 2011; Porras et al. 2017). The Dutch experiences of addressing ecosystem services in commodity value chains shows that this has often been problematic, with effectiveness being highly dependent upon the framework conditions, notably macro-political support, trade and cultural values, and the willingness to change from government regulatory arrangements to market arrangements (Savilaakso et al. 2015). Such efforts and also riddled with thorny questions of power, legitimacy, inclusiveness and participation, efficiency and efficacy (Waarts and Basso Gumbis de Souza 2017), in common with similar experiences internationally (Pagiola et al. 2005; Bulte et al. 2008; Pirard et al. 2010). These insights suggest that that compatibility in landscape and value chain governance is needed to achieve sustainable value chains, as was noted particularly in the PES, REDD+ and ForCES cases where ecosystem services were most explicit. This includes dealing with trade-offs, maximizing overlaps and combining separate policy instruments and governance arrangements which are typically unconnected and often opposing to link ecosystem and poverty reduction agendas (Porras et al. 2017).

Despite the obvious links between sustainable value chains and landscapes, only recently have explicit policies and governance arrangements emerged which seek to integrate ecosystem services into value chains and take an integrated landscape approach. The only Dutch policy referring to both landscapes and ecosystems is the Natural Capital Agenda. Action point five states the need to address conservation in agro-commodity production areas from a landscape level, with a landscape approach and integrated land-use planning suggested as beneficial as it *“will help create larger areas of valuable, protected nature, to replace the present fragmented landscape of small areas protected at farm level”* (Netherlands Government 2013, p 6). Landscape approaches have been promoted by IDH since 2015 as part of its second strategy sustainable trade (IDH 2016), which implements the Natural Capital Agenda through a series of co-financed public-private-CSO partnerships in 11 landscapes worldwide, which link with the sourcing areas of IDHs commodity value chain orientated programs (IDH 2017). Furthermore, landscape approaches are also a governance arrangement (Hospes et al. 2016). The Dutch Environment Agency now recognizes this (Van der Horn and Meijer 2015), implying that more interlinks between value chain and landscape governance appear to be appropriate.

Whilst commodities have been recognized as a source of competing land use (Giller et al. 2008; Nelson et al. 2009; Sayer et al. 2013), Sayer and colleagues 10 principles for an integrated landscape approach do not elaborate on the interlinkages and benefits between sustainable value chains and the origin landscapes of these products. It is now increasingly realized that some sustainability goals are only possible at landscape level, and thus require inter-sectoral geographical coordination (Mbow et al. 2015; Reed et al. 2015; Waarts and Basso Gumbis de Souza 2017). Advancing knowledge on sustainable commodity chains and integrated landscape approaches highlights the need for a more seamless understanding of the landscape-scale complexity of our production systems (Giovannucci et al. 2014) and the benefits of an integrated landscape approach (Muller et al. 2010; Ros-Tonen et al. 2015; van Oosten et al. 2017; Deans et al. 2017; Arts et al. 2017). The fact that communication with the public sector in sector-based round-tables and platforms tends to be restricted to only one ministry in the production countries, hampers effective and integrated implementation of government policies in origin landscapes (Waarts and Basso Gumbis de Souza 2017).

Based on these findings, the following suggestions were made by interviewees and by the authors, of options to further integrate sustainable use and maintenance of ecosystem services into tropical commodity value chains, directed toward all stakeholders in the value chains (business, government, CSOs and support):

(i) Value chain actors need to collaborate to develop clear, coherent and integrated strategies that make the role of ecosystem services and landscapes in global commodity value chains explicit in policy concepts such as “sustainable inclusive growth”, “natural capital”, and “green economic growth”;
(ii) Governments in consumer countries can re-consider the mix of policy instruments, using market governance certification “carrots” and incentive-based “sticks” (such as tax incentives and public procurement) to stimulate new partnerships and initiatives with regulation to ensure full chain, sector and landscape coverage to defined standards;
(iii) Governments can more explicitly create standards for ecosystem services in their procurement criteria to stimulate how these services are addressed in origin landscapes;
(iv) Governments in end-of-chain countries can re-consider the mix of policy instruments used, expanding from the focus on voluntary product certification to alternative instruments which support the private sector to respond to market opportunities for ecosystem services, for example incentivizing demand through fiscal incentives;
(v) Governments from sourcing regions and consumer countries should jointly discuss with standards organizations how a more explicit inclusion of ecosystem goods and services in the landscapes from which they originate can be addressed in voluntary sustainability standards;
(vi) Bringing together stakeholders with a landscape focus (e.g., the UN FAO and Global Landscapes Forum) and commodity focus (e.g., the UNDP Green Commodities Program) and voluntary standard platforms such as the ISEAL Alliance, operating at multiple levels where value chains and landscapes intersect, could also better integrate ecosystem services into the different segments of a chain more effectively;
(vii) Making use of the interconnections between products and experiences across chains and origin landscapes could provide valuable multi-level, cross-sectoral information, as could government involvement in certification schemes—such as FSC and UTZ—which take a “beyond the chain” integrated landscape approach, to provide insights for all types of chain actors, particularly, consumers, origin country governments and smaller enterprises) into the costs and benefits of maintaining ecosystem services as part of sustainable chains and origin landscapes;
(viii) Sectoral ministries should ensure a coherent approach when using indirect policy tools that facilitate and endorse ecosystems services to take an integrated landscape approach;
(ix) Policy possibilities should be explored to influence how ecosystem services are impacted in other stages in the chain than the current focus on origin countries. The processing and consumption stages which take place in end-of-chain countries where the sphere of influence and range of policy options available is greater for consumer country governments such as the Netherlands;
(x) Consumer and origin country governments should increase collaboration to develop, implement and enforce policies and governance that stimulates sustainable production practices;
(xi) European processing and consumer country governments could collaborate more to address sustainable chains, both bilaterally and regionally (the European Union) to ensure a level playing field and to stimulate demand for products which address ecosystem services all along their value chains and not just in the sourcing landscape;
(xii) More evidence is needed of the impact of governance arrangements and particularly certification at landscape level and how it maintains or enhances ecosystem services, with internationally agreed upon impact indicators.

## Conclusions

Over the past decade, policymakers in the Netherlands together with private sector and societal organizations, have experimented with different governance arrangements to address the sustainability of international commodity value chains with strong Dutch links. The result has been rapidly shifting policies and on-the-ground practices relating to sustainability of the value chains in general, and increased attention to enhancing the maintenance of ecosystem services specifically. Challenges have included making ecosystem services explicit in policy and governance arrangements and engaging with all value chain actors, particularly consumers and origin country governments, with all types of private sector actors. The cases highlight that organizing multi-actor involvement in international value chains is seen as a critical factor for the success and acceptance of sustainability initiatives addressing ecosystem services, such as voluntary standards and certification.

The Dutch cases show how the government created incentives for actors to address ecosystem services in international commodity value chains politically feasible, using endorsing, facilitating and partnership policies. However, the main approach used—market governance—has had limits in terms of effectiveness, sector uptake and impact to date, with the business incentives to invest in internalizing these concepts not clear for all private sector actors. Chains sourced in tropical landscapes may be governed more effectively for sustainability if the voluntary, market policy tools and governance arrangements used have more integrated goals that take account of sourcing landscapes and impacts along entire value chain. Given the limitations of market governance, governance arrangements that more fully support the transition to value chains that address the entire range of ecosystem services impacted by commodity production and trade and create a level playing field for all market players are needed. Scoones et al. (2015) stress that green transformations must be both “top-down”, involving elite alliances between governments and business, but also ‘bottom-up’, pushed by grassroots innovators and entrepreneurs, and part of wider mobilizations among civil society.

It is increasingly recognized that we need to go further and go one step beyond current policies and governance practices: “Beyond certification” (Barry 2015; Poynton 2015) to certification plus and “beyond the chain” (Ros-Tonen et al. 2015). By integrating landscape and value chain governance, ecosystems and their services extending from the sourcing landscape in which the value chain originates to the end of its chain, can be addressed to create more sustainable commodity value chains. Both value chain and landscape approaches pursue environmental, social and economic sustainability. Both governance approaches focus on integrated objectives, multi-stakeholder, multi-level arrangements and learning processes to achieve “sustainable landscapes” (i.e., the sourcing area) (Sayer et al. 2013; van Oosten et al. 2014; Ros-Tonen et al. 2014; Reed et al. 2016). These intersections and common issues of concern suggest that there is scope for increased alignment. A “landscape + value chain” approach appears possible, although evidence on how this works in practice is as yet thin (Waarts and Basso Gumbis de Souza 2017). Benefits could include a more coherent and integrated approach to the regions where tropical commodities are sourced, that addresses both the social and economic aspects of the people and organizations involved in the value chains, and the environmental sustainability of the commodity by systematically considering ecosystem services provided by the landscape. Recognizing the difficulties inherent in both landscape and value chain approaches is critical. These include issues of inclusiveness (Helmsing and Vellema 2011; Ros-Tonen et al. 2015) so that ecosystem and societal and business benefits and externalities are balanced; the need to take an adaptive learning approach to effectively implement such complex governance arrangements (van Oosten 2013) and the importance of actively seeking synergistic, complementarity between government regulation and market governance (Gulbrandsen 2014; Ingram 2014). As landscape approaches to date often have not made governance explicit, but have been presented as management processes (Hospes et al. 2016), recognizing that both value chain approaches and landscape approaches are forms of governance is also key to any approach seeking to enhance ecosystem services in cocoa, soy, tropical timber and palm oil value chains.
